# Using conventional *F*-statistics to study unconventional sex-chromosome differentiation

**DOI:** 10.7717/peerj.3207

**Published:** 2017-04-27

**Authors:** Nicolas Rodrigues, Christophe Dufresnes

**Affiliations:** Department of Ecology & Evolution, University of Lausanne, Lausanne, Switzerland

**Keywords:** *Hyla arborea*, *Rana temporaria*, Sex determination, Population genetics, *F*_is_, *F*_st_, Microsatellites, Population genomics, Homomorphic sex chromosomes, Sex-linked markers

## Abstract

Species with undifferentiated sex chromosomes emerge as key organisms to understand the astonishing diversity of sex-determination systems. Whereas new genomic methods are widening opportunities to study these systems, the difficulty to separately characterize their X and Y homologous chromosomes poses limitations. Here we demonstrate that two simple *F*-statistics calculated from sex-linked genotypes, namely the genetic distance (*F*_st_) between sexes and the inbreeding coefficient (*F*_is_) in the heterogametic sex, can be used as reliable proxies to compare sex-chromosome differentiation between populations. We correlated these metrics using published microsatellite data from two frog species (*Hyla arborea*and *Rana temporaria*), and show that they intimately relate to the overall amount of X–Y differentiation in populations. However, the fits for individual loci appear highly variable, suggesting that a dense genetic coverage will be needed for inferring fine-scale patterns of differentiation along sex-chromosomes. The applications of these *F*-statistics, which implies little sampling requirement, significantly facilitate population analyses of sex-chromosomes.

## Introduction

In sharp contrast with the classical sex-determining systems of mammals and birds, the study of sex-chromosome evolution in other vertebrate lineages has revealed a myriad of alternative evolutionary trajectories (Beukeboom & Perrin, 2014). Species with homomorphic gametologs are providing instrumental insights into the mechanisms paving these unconventional pathways, like the rates of sex-chromosome transitions (e.g., Dufresnes et al., 2015), the dynamics of X–Y recombination (e.g., Stöck et al., 2013; Dufresnes et al., 2014b), the evolution of X–Y differentiation (e.g., Yoshida et al., 2014), as well as the interplay between genetic and non-genetic sex-determination (e.g., Rodrigues et al., 2015; Perrin, 2016). Often neglected due to the lack of genomic resources, these promising non-model organisms can now be widely exploited for sex-chromosome research with low-cost population genomic techniques (Brelsford, Dufresnes & Perrin, 2016a; Brelsford et al., in press). However, given the rapid evolution of the forces at work, patterns of variation at sex-linked markers can be complex and population-specific (Rodrigues et al., 2014; Dufresnes et al., 2014a; Dufresnes et al., 2014b), prompting for multilevel analyses in order to get comprehensive inferences.

A key variable to such analyses is the amount of differentiation between sex chromosomes. This feature, central to the evolutionary history of sex chromosomes, is highly informative regarding their contribution to sex-determination, how they differentiate and which genomic regions are affected. For instance, mapping peaks of X–Y divergence can point to sex-determining regions (e.g., Brelsford, Dufresnes & Perrin, 2016b); in a similar fashion, it can be used to screen for sex-antagonistic genes and thus test their hypothetical role in triggering the suppression of X–Y recombination (Kirkpatrick & Guerrero, 2014), a critical and criticized assumption in the sex-chromosome literature (Beukeboom & Perrin, 2014; Wright et al., 2016).

Measuring sex-chromosome differentiation in species with “undifferentiated” sex chromosomes is by definition challenging. Unlike in mammals and birds, these sex chromosomes are largely homologous. Thus, estimating genetic divergence between the X and Y copies of homologous loci requires their separate genotyping (by cloning methods), or to phase X and Y haplotypes in males from patterns of linkage disequilibrium. Both of these approaches have severe limitations for population genetics and phylogeographic analyses. Cloning is only adequate for genotyping few genes in few individuals. Phasing diploid genotypes requires tremendous sampling and genotyping efforts, including large adult (males and females) and family samples (crosses) in populations. Moreover, given that it relies on linkage disequilibrium, the latter is easier and thus biased towards populations where XY recombination is low or null (and XY differentiation is high). Already challenging with small datasets like microsatellite genotypes, haplotype reconstruction becomes a struggle with high-throughput genomic data.

An indirect ad hoc alternative is to compute allele frequency indices on sexed samples, like *F*-statistics. Genetic distance between males and females from a panmictic population should be proportional to the amount of X–Y differentiation. Because males share half of their sex-linked alleles with females (the X copies), pairwise *F*_st_ between sexes (♂–♀*F*_st_) is thus expected to span from 0.0 (null X–Y differentiation) to 0.5 (complete X–Y differentiation). Even simpler, X–Y differentiation can theoretically be quantified through the excesses of heterozygotes at sex-linked loci in the heterogametic sex, i.e., XY males, thus without the systematic need for female samples. Heterozygote excess is commonly depicted by negative *F*_is_ values. Hence, male *F*_is_ (♂*F*_is_) at sex-linked loci should span from 0.0 (no X–Y differentiation) to −1.0 (complete X–Y differentiation) in populations at Hardy–Weinberg Equilibrium (HWE). The rationales of these ad hoc approaches appear straightforward and have been used in few previous studies (e.g., Shikano et al., 2011; Natri, Shikano & Merilä, 2013; Dufresnes et al., 2014b; Rodrigues et al., 2014). However, these *F*-statistics may also be influenced by other processes such as sex-specific dispersal, departure from HWE due to demographic processes, as well as drift shaping marker-specific signals, all of which may temper their reliability to estimate sex-chromosome differentiation. Thus, encouraging their application first necessitates proper assessment in comprehensive population genetic frameworks.

Here we demonstrate the informativeness of ♂–♀*F*_st_ and ♂*F*_is_ at sex-linked markers to reliably compare sex-chromosome differentiation between natural populations. We extracted and correlated these statistics from published microsatellite datasets of two famous study systems in the field of sex determination: the male-heterogametic frogs *Hyla arborea* and *Rana temporaria*, for which data from multiple populations are available for such comparison. The little requirements of these methods significantly enlarge opportunities for the study of homomorphic sex chromosomes in a wide array of non-model organisms.

## Methods

### *Hyla arborea* data

This dataset includes sex-linked microsatellite genotypes across the entire range of the species in Europe, used to understand the evolution of X–Y differentiation and recombination in a phylogeographic framework (Dufresnes et al., 2014b; dryad doi: http://dx.doi.org/10.5061/dryad.45j84). To this end, using male and female adult samples (distinguished based on secondary sexual traits, i.e., the presence/absence of vocal sacs on the throat), combined with family data (parents + offspring), the authors could phase X and Y haplotypes for 11 microsatellite loci (details in Dufresnes et al., 2014b) across 28 populations of at least 5 males, and computed a metric of X–Y differentiation based on allele frequency overlap (described in Dufresnes et al., 2014b; page 3447). We extracted this data and computed ♂*F*_is_ for these populations using FSTAT (Goudet, 1995). We also calculated *F*_st_ between sexes (♂–♀*F*_st_) for a subset of 14 of these populations, where at least five individuals of each sex were available (Table S1A). Sample size of less than five individuals were not considered in order to include only statistically robust estimates.

Moreover, in order to account for the baseline levels of inbreeding (see ‘Results & Discussion’), we estimated the *F*_is_ of females at sex-linked loci (♀*F*_is_). For the same purpose, we mined a second published dataset to compute *F*_is_ from autosomal microsatellite genotypes (autosomal *F*_is_), which are available for 27 out of the 28 populations (Dufresnes et al., 2013; dryad doi: http://dx.doi.org/10.5061/dryad.2vk30; 30 loci). We then adjusted ♂*F*_is_ by computing the difference with either ♀*F*_is_ or autosomal *F*_is_.

For each comparison, we fitted linear regression models in R (R Core Team, 2016).

### Rana temporaria data

This dataset includes microsatellite genotypes (11–13 loci) of the sex-linkage group from six Swedish and four Swiss populations of at least five individuals of each sex (Rodrigues et al., 2013; dryad doi: http://dx.doi.org/10.5061/dryad.0mg7h; Rodrigues et al., 2014; dryad doi: http://dx.doi.org/10.5061/dryad.mb06v). This data was originally generated to investigate levels of sex-specific genetic differentiation at this linkage group to assess the relative contribution of genetic *vs.* non-genetic components of sex-determination in this species. As for *H. arborea*, we computed ♂*F*_is_, ♂–♀*F*_st_ as well as ♀*F*_is_ for each population (Table S1B), and fitted linear regression models. However, no measure of X–Y differentiation nor autosomal variation is available for these populations.

## Results & Discussion

**Figure 1:**
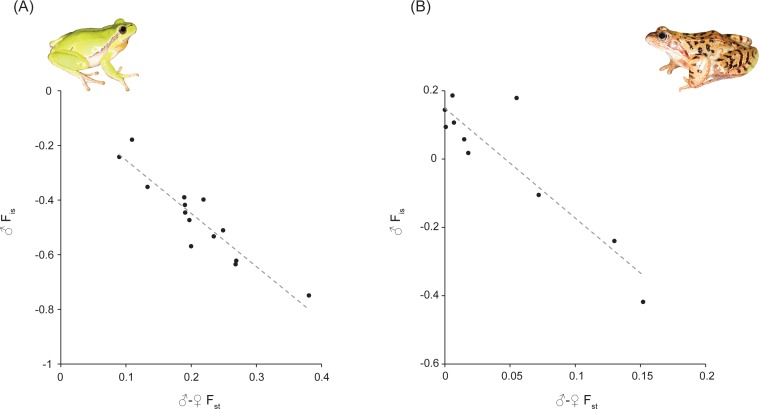
*F*_*st*_ between sexes (♂–♀*F*_st_) versus male *F*_is_ (♂*F*_is_) at sex-linked loci in *Hyla arborea* and *Rana temporaria*. Both are highly significant (Table 1). Photo credit: Christophe Dufresnes.

**Figure 2:**
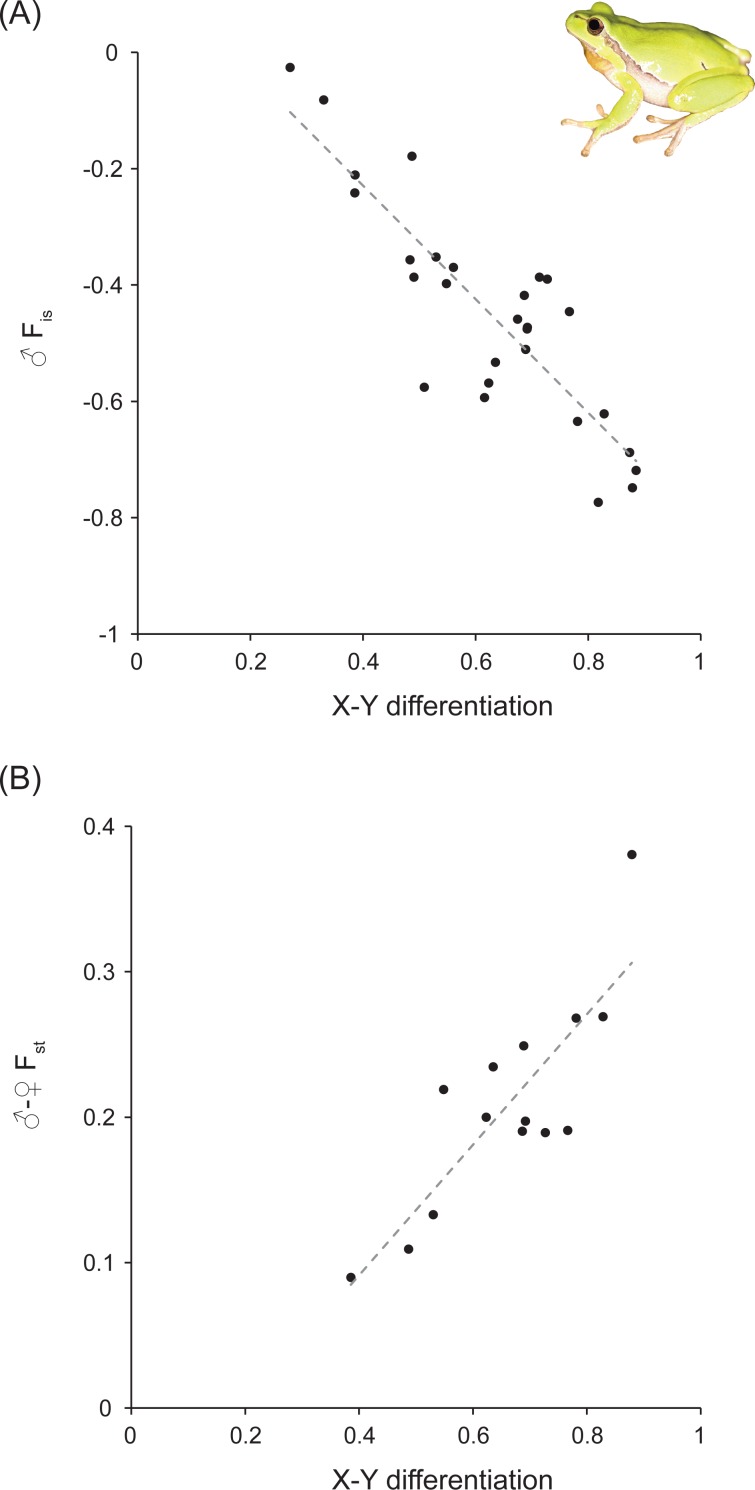
X–Y differentiation versus male *F*_is_ (♂*F*_is_) and *F*_st_ between sexes (♂–♀*F*_st_) at sex-linked loci in *Hyla arborea*. Both are highly significant (Table 1). Photo credit: Christophe Dufresnes.

We established significant correlations between the different statistics for both species (Fig. 1 and Table 1). As expected, ♂*F*_is_ is negatively correlated with *F*_st_ between sexes (for *H. arborea*: *R*^2^ = 0.86; for *R. temporaria*: *R*^2^ = 0.82). Moreover, for *H. arborea*, we can further show that these two estimates are well-correlated with a measure of X–Y differentiation computed from phased genotypes (for ♂*F*_is_: *R*^2^ = 0.75;  for ♂–♀*F*_st_: *R*^2^ = 0.71; Fig. 2 and Table 1). Thus, both statistics appear as reliable proxies to estimate overall differentiation between sex chromosomes.

**Table 1:** Correlation between male *F*_is_ (♂*F*_is_), *F*_st_ between sexes (♂–♀*F*_st_) and X–Y differentiation (X–Y dif.) at sex-linked loci. ♂*F*_is_ was also adjusted by *F*_is_ at autosomal loci (auto. *F*_is_) and *F*_is_ at sex-linked loci in female (♀*F*_is_).

	*H. arborea*			*R. temporaria*		
	*N*	*R*^2^	*P*	*N*	*R*^2^	*P*
♂*F*_is_ *vs*. ♂–♀*F*_st_	14	0.86	<0.001	10	0.82	<0.001
♂*F*_is_ (adjusted by auto. *F*_is_) *vs*. ♂–♀*F*_st_	14	0.86	<0.001	–	–	–
♂*F*_is_ (adjusted by ♀*F*_is_) *vs*. ♂–♀*F*_st_	14	0.70	<0.001	10	0.90	<0.001
♂–♀*F*_st_*vs.* X–Y dif.	14	0.71	<0.001	–	–	–
♂*F*_is_ *vs*. X–Y dif.	28	0.75	<0.001	–	–	–
♂*F*_is_ (adjusted by auto. *F*_is_) *vs*. X–Y dif.	27	0.70	<0.001	–	–	–
♂*F*_is_ (adjusted by ♀*F*_is_) *vs*. X–Y dif.	14	0.43	0.010	–	–	–

**Notes.**

Abbreviations N number of populations *R*^2^ fit of linear regression *P* *p*-value of linear regressions

However, we further report strong variation among the individual fits of each locus in both species (Figs. S1 and S2). The *R*^2^ associated with the regressions of ♂*F*_is_ by ♂–♀*F*_st_ averaged 0.54 ± 0.32 for *H. arborea* (Fig. S1) and 0.57 ± 0.33 for *R. temporaria* (Fig. S2). Although lower sample sizes may account for part of this variation (as some loci were not informative in every populations), such fluctuations may also likely be due by stochastic processes like drift. Thus, at least several markers appear needed to obtain sound estimations. While this is usually the case for studies of whole-chromosome differentiation (e.g., Dufresnes et al., 2014a; Dufresnes et al., 2014b), it might become an issue for comparing fine-scale patterns along chromosomal segments (e.g., sliding window analyses), which then requires a denser coverage to obtain meaningful estimates.

The ♂*F*_is_ statistic is also expected to be affected by the baseline level of inbreeding in populations. Here it should not have impacted the comparisons for *H. arborea*, since the populations analyzed are known to meet Hardy–Weinberg Equilibrium (HWE), as inferred from autosomal markers (Dufresnes et al., 2013). Accordingly, controlling ♂*F*_is_ by autosomal *F*_is_ yielded similarly good correlations (Table 1, Fig. S1). In parallel, we also tested whether *F*_is_ at sex-linked markers in females (♀*F*_is_) could be used for the adjustments instead, in absence of autosomal data. The resulting fits were quite variable, being overall better for *R. temporaria*, but worse for *H. arborea* (Table 1, Figs. S1 and S2). These inconsistencies may indicate that ♀*F*_is_ is a poor corrector for such analysis. One explanation probably lies within the effective size of X chromosomes, which depends on their amount of recombination with the Y, i.e., $ \frac{3}{4} $ of autosomes if X–Y recombination is suppressed, but similar to autosomes if both copies freely recombine. Here it should strongly fluctuate among the different populations considered, given their contrasted sex-chromosome dynamics. In *H. arborea*, X–Y recombination rates were shown to evolve rapidly and strongly vary between populations (Dufresnes et al., 2014a; Dufresnes et al., 2014b). In *R. temporaria*, sex-determination is not strictly genetic, and so the same loci behave either like non-recombining sex chromosomes, or autosomes, depending on populations (Rodrigues et al., 2014; Rodrigues et al., 2015; Rodrigues et al., 2016). In parallel, sex-biased dispersal may also account for such discrepancies, by inflating *F*_is_ of the dispersing sex (i.e., towards a larger heterozygote deficit, Goudet, Perrin & Waser, 2002). Some evidence did suggest sex-biased dispersal in our focal species, i.e., male-biased in *H. arborea* (based on capture-mark-recapture data; Vos, Ter Braak & Nieuwenhuizen, 2000) but female-biased in *R. temporaria* (based on genetic data; Palo et al., 2004). Therefore, given our results and the potential cofounding factors affecting sex-specific *F*_is_, autosomal *F*_is_ (ideally computed from samples of both sexes) should thus rather be considered to correct sex-linked ♂*F*_is_, whenever possible. Moreover, allele dropout, which is inherent to some commonly used genotyping-by-sequencing methods like RAD (Restriction site-associated DNA), can lead to overestimate *F*_is_ (Gautier et al., 2013). However, this process being likely random, it should similarly affect autosomal and sex-linked markers; ♂*F*_is_ relative to autosomal *F*_is_ should thus be comparable among populations.

The low sampling requirement for computing these *F*-statistics significantly simplifies population genetic analyses of homomorphic sex-chromosomes. *F*_st_ between sexes was used to this purpose in our previous studies to investigate the geographic patterns of sex-chromosome differentiation (Rodrigues et al., 2013; Rodrigues et al., 2014; Dufresnes et al., 2014b), with coherent results. Moreover, sex-linked ♂*F*_is_, was also successfully applied in studies of sex-chromosome differentiation in stickleback fishes (Shikano et al., 2011; Natri, Shikano & Merilä, 2013). Importantly, ♂*F*_is_ has the advantage not to rely on female genotypes, which are usually the conspicuous sex and are thus harder to sample in many species. This metric actually opens opportunities to exploit sample series that were not originally designed for sex-chromosome studies (e.g., museum collections), and where a majority of males is represented. Furthermore, these approaches should also be applicable to female-heterogametic systems (ZW), by computing ♀*F*_is_. In fact, due to the high recombination rates usually observed in females (Brelsford, Dufresnes & Perrin, 2016a; Brelsford, Rodrigues & Perrin, 2016), reconstructing Z and W haplotypes may be virtually impossible, so ♀*F*_is_ and ♂–♀*F*_st_ would be the only way to compare Z–W differentiation between populations. Combining these simple statistics with population genomic data will guarantee exciting new insights into the unusual ways sex chromosomes evolve in many organisms.

##  Supplemental Information

10.7717/peerj.3207/supp-1 Table S1 Details on the data analyzed

10.7717/peerj.3207/supp-2 Figure S1 *F*_*st*_ between sexes (♂–♀*F*_*st*_) versus male *F*_*is*_ (♂*F*_*is*_) for each sex-linked locus in *Hyla arborea*

10.7717/peerj.3207/supp-3 Figure S2 *F*_*st*_ between sexes (♂–♀*F*_*st*_) versus male *F*_*is*_ (♂*F*_*is*_) for each sex-linked locus in *Rana temporaria*

## Additional Information and Declarations

### Data Availability

The following information was supplied regarding data availability: The raw data originates from published studies. Statistics used in this study are available as Table S1.
