# Supplementary material for: Using conventional F-statistics to study unconventional sex-chromosome differentiation
Source: PeerJ. 2017 Apr 27;5:e3207. doi: 10.7717/peerj.3207 (PMC5410149; doi:10.7717/peerj.3207)
Supplement: Table S1 [file peerj-05-3207-s001.pdf]

Supporting Information

Table S1a: Summary of the *Hyla arborea* data used in this study, extracted from Dufresnes et al. (2014b) for sex-linked markers and Dufresnes et al. (2013) for autosomal data (30 loci). ID: locality number used in the mentionned publications; N_m: number of male samples; N_f: number of female samples; X-Y dif.: X-Y differentiation estimated by Dufresnes et al. (2014b); N: number of samples.

		sex-linked loci						autosomal loci	
ID	Country	N_m	N_f	♂-♀ F_{st}	♂ F_{is}	♀ F_{is}	X-Y dif.	N	F_{is}
1	Greece	25	27	0.09	-0.24	0.02	0.39	52	0.01
2	Greece	6	-	-	-0.08	-	0.33	6	0.02
3	Greece	25	-	-	-0.03	-	0.27	25	0.09
5	Greece	20	-	-	-0.21	-	0.39	22	-0.02
8	Greece	18	7	0.13	-0.35	-0.06	0.53	25	0.09
9	Greece	18	-	-	-0.36	-	0.48	18	0.09
11	Greece	14	-	-	-0.39	-	0.49	16	0.06
24	Serbia	6	-	-	-0.37	-	0.56	7	0.10
28	Albania	8	10	0.11	-0.18	-0.14	0.49	20	-0.01
30	Croatia	18	-	-	-0.59	-	0.62	18	0.06
31	Croatia	7	5	0.20	-0.57	0.12	0.62	12	-0.09
32	Croatia	20	-	-	-0.58	-	0.51	22	0.03
33	Croatia	14	12	0.23	-0.53	0.03	0.64	26	0.07
37	Croatia	18	18	0.22	-0.40	-0.01	0.55	36	0.02
38	Croatia	25	25	0.25	-0.51	0.05	0.69	50	0.07
39	Croatia	19	-	-	-0.48	-	0.69	19	0.06
40	Serbia	15	14	0.19	-0.42	-0.07	0.69	30	0.00
41	Romania	11	-	-	-0.46	-	0.67	11	0.01
42	Romania	5	6	0.19	-0.39	-0.05	0.73	13	0.02
43	Romania	10	8	0.19	-0.45	0.00	0.77	20	0.07
44	Hungary	15	-	-	-0.39	-	0.71	16	0.00
45	Austria	18	8	0.20	-0.47	-0.20	0.69	26	0.03
48	Poland	9	-	-	-0.69	-	0.87	9	0.08
49	Switzerland	18	15	0.27	-0.62	0.06	0.83	33	0.05
55	Netherlands	19	-	-	-0.72	-	0.89	23	-0.05
56	Switzerland	15	15	0.38	-0.75	0.16	0.88	30	0.07
57	France	22	7	0.27	-0.64	-0.23	0.78	29	-0.02
66	Sweden	8	-	-	-0.77	-	0.82	-	-

Table S1b: Summary of the *Rana temporaria* data used in this study, extracted from Rodrigues et al. (2013, 2014). N_m: number of male samples; N_f: number of female samples.

		sex-linked loci				
Locality	Country	N_m	N_f	♂-♀ F_{st}	♂ F_{is}	♀ F_{is}
Esrange	Sweden	24	28	0.02	0.02	-0.03
Ammarnäs	Sweden	24	21	0.15	-0.42	0.03
Hampjärn-Grytan	Sweden	27	20	0.13	-0.24	0.03
Häggedal	Sweden	28	23	0.07	-0.11	0.09
Lindragen	Sweden	16	9	0.06	0.18	0.40
Tvedöra	Sweden	22	23	0.01	0.11	0.06
Lavigny	Switzerland	18	18	0.00	0.09	0.05
Cossonay	Switzerland	6	6	0.00	0.14	0.10
Bex	Switzerland	31	31	0.01	0.06	0.02
Retaud	Switzerland	5	5	0.01	0.19	0.06
