# Supplementary material for: Using conventional F-statistics to study unconventional sex-chromosome differentiation
Source: PeerJ. 2017 Apr 27;5:e3207. doi: 10.7717/peerj.3207 (PMC5410149; doi:10.7717/peerj.3207)

Figure S1: F_{st} between sexes (♂-♀ F_{st}) versus male F_{is} (♂ F_{is}) without correction (A), corrected by female F_{is} (♀F_{is}) (B) or autosomal F_{is} (C) for each sex-linked loci in *Hyla arborea*. R^2 of the linear regressions are indicated, and a regression line is shown when the regression was significant. Photo credit: Christophe Dufresnes

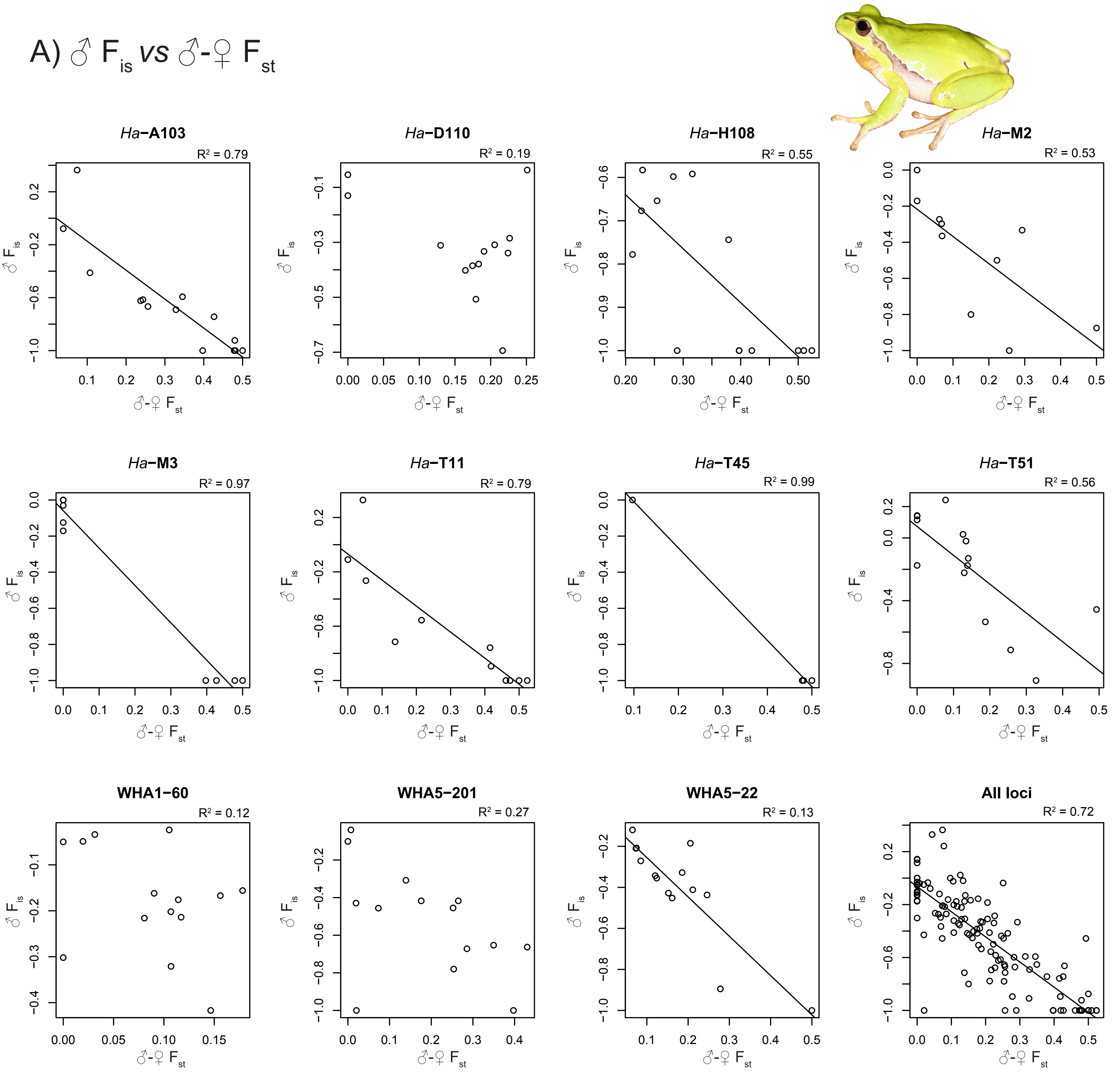

A) ♂ Fis vs ♂-♀ Fst
Ha–A103
R² = 0.79
Ha–D110
R² = 0.19
Ha–H108
R² = 0.55
Ha–M2
R² = 0.53
Ha–M3
R² = 0.97
Ha–T11
R² = 0.79
Ha–T45
R² = 0.99
Ha–T51
R² = 0.56
WHA1–60
R² = 0.12
WHA5–201
R² = 0.27
WHA5–22
R² = 0.13
All loci
R² = 0.72
♂ Fis
♂-♀ Fst

B) ♂ F_{is} (corrected by ♀ F_{is}) *vs* ♂-♀ F_{st}

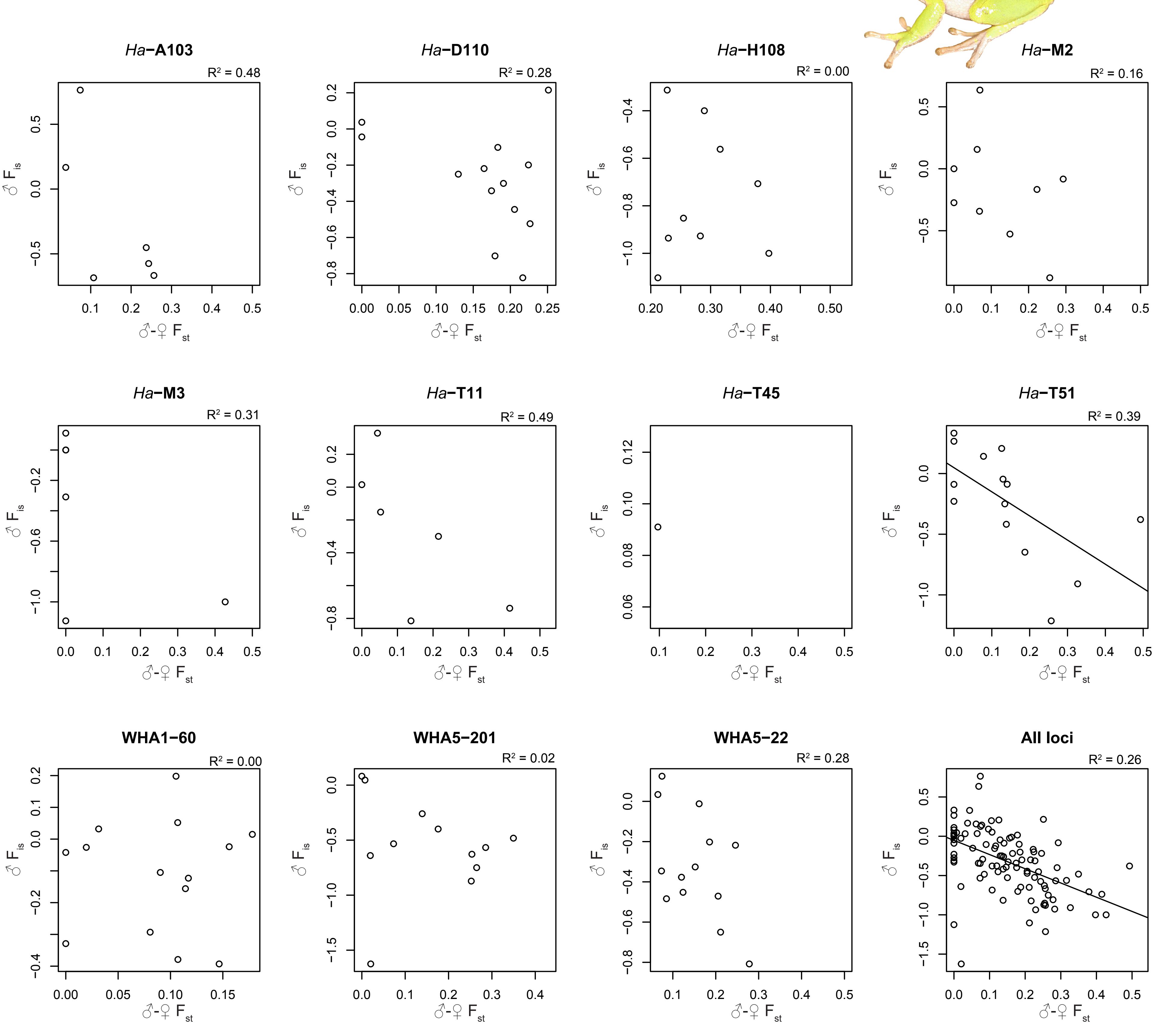

C) ♂ F_{is} (corrected by autosomal F_{is}) *vs* ♂-♀ F_{st}

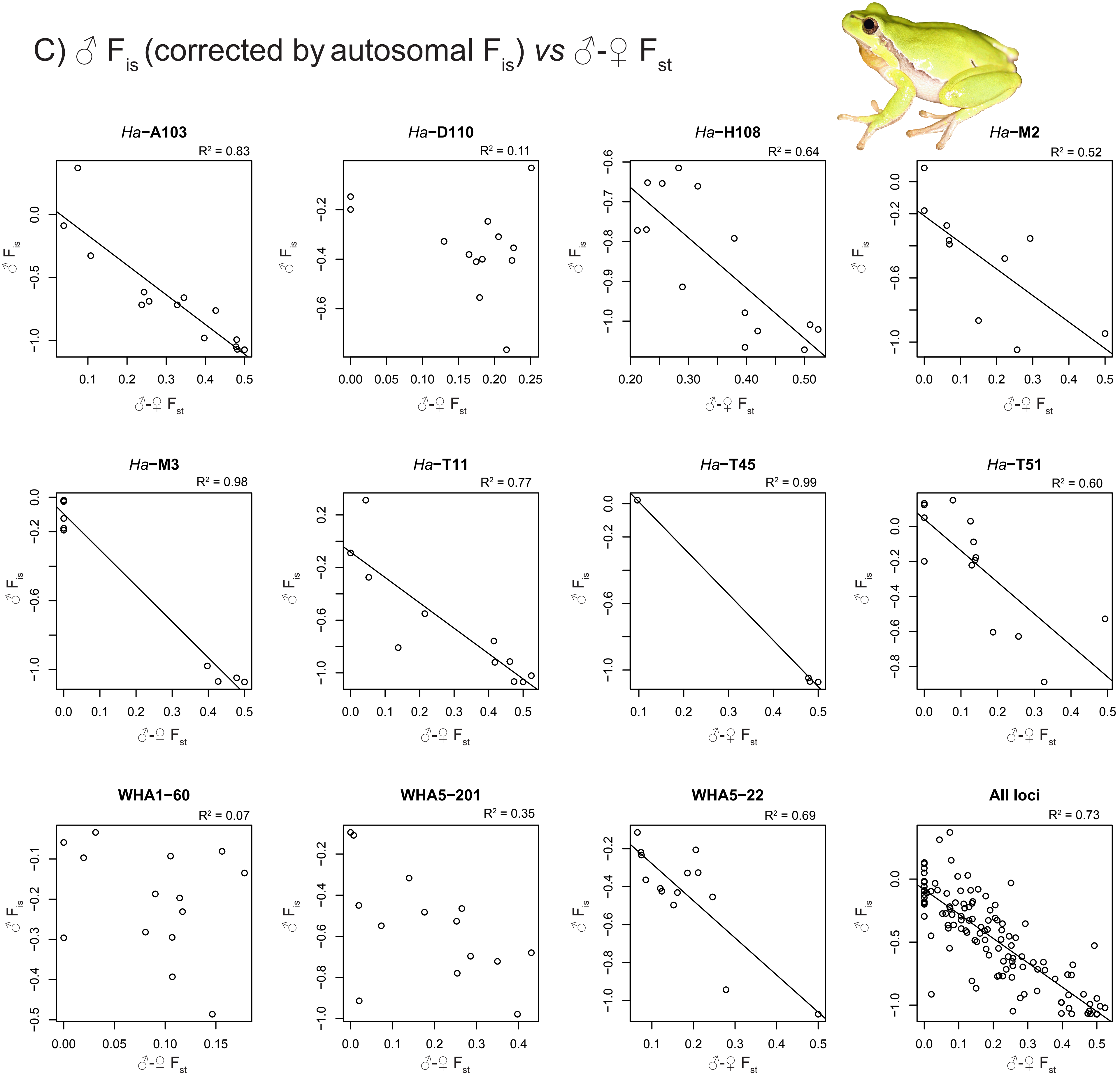

Supplement: Figure S1 [file peerj-05-3207-s002.pdf]
