# Supplementary material for: Using conventional F-statistics to study unconventional sex-chromosome differentiation
Source: PeerJ. 2017 Apr 27;5:e3207. doi: 10.7717/peerj.3207 (PMC5410149; doi:10.7717/peerj.3207)

Figure S2: F_{st} between sexes (♂-♀ F_{st}) versus male F_{is} (♂ F_{is}) without correction (A) or corrected by female F_{is} (♀F_{is}) (B) for each sex-linked loci in *Rana temporaria*. R^2 of the linear regressions are indicated, and a regression line is shown when the regression was significant.
Photo credit: Christophe Dufresnes

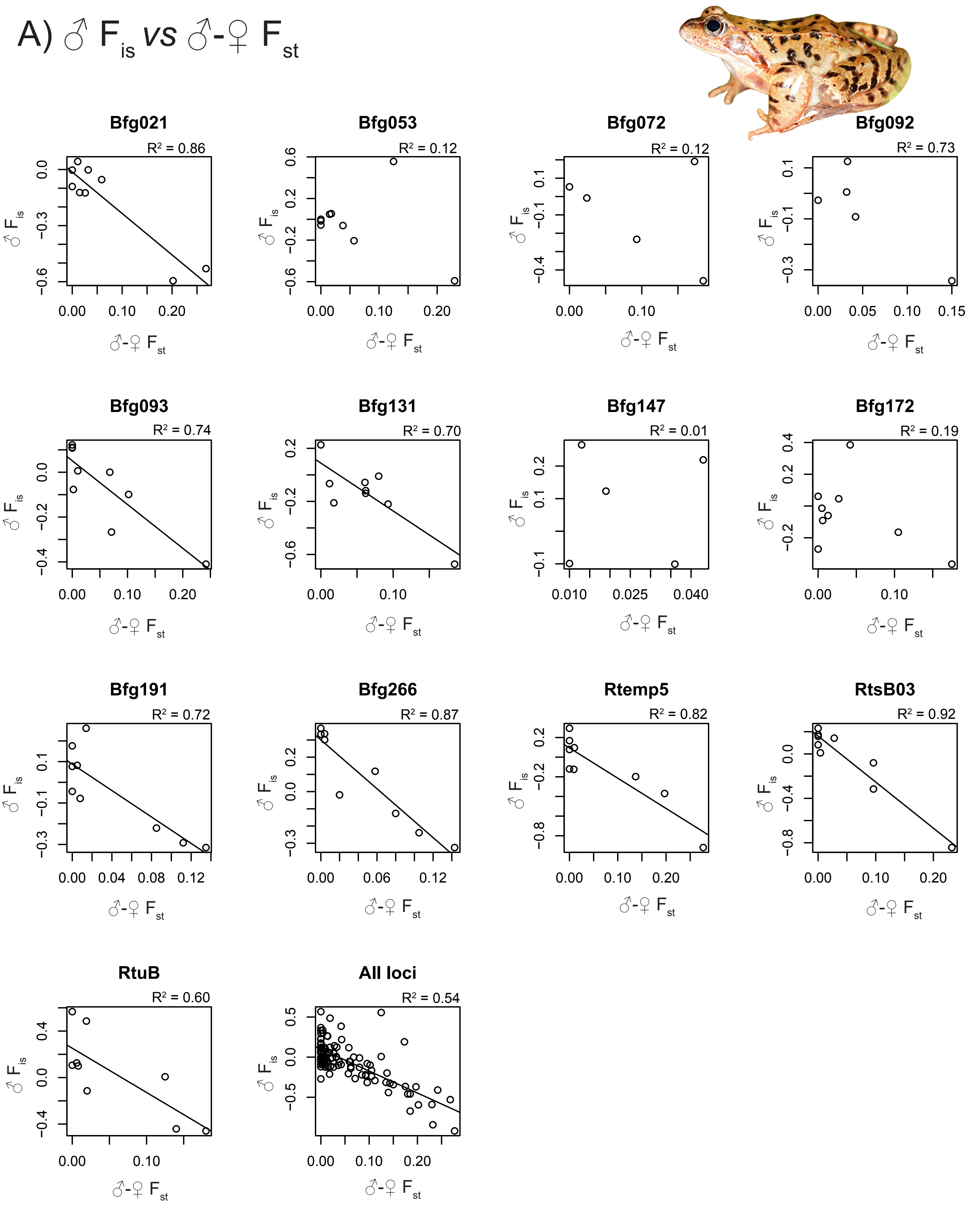
A) ♂ Fis vs ♂-♀ Fst
Bfg021
R² = 0.86
Bfg053
R² = 0.12
Bfg072
R² = 0.12
Bfg092
R² = 0.73
Bfg093
R² = 0.74
Bfg131
R² = 0.70
Bfg147
R² = 0.01
Bfg172
R² = 0.19
Bfg191
R² = 0.72
Bfg266
R² = 0.87
Rtemp5
R² = 0.82
RtsB03
R² = 0.92
RtuB
R² = 0.60
All loci
R² = 0.54
♂ Fis
♂-♀ Fst

B) ♂ F_{is} (corrected by ♀ F_{is}) *vs* ♂-♀ F_{st}

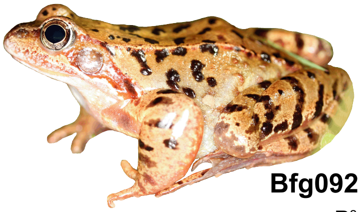

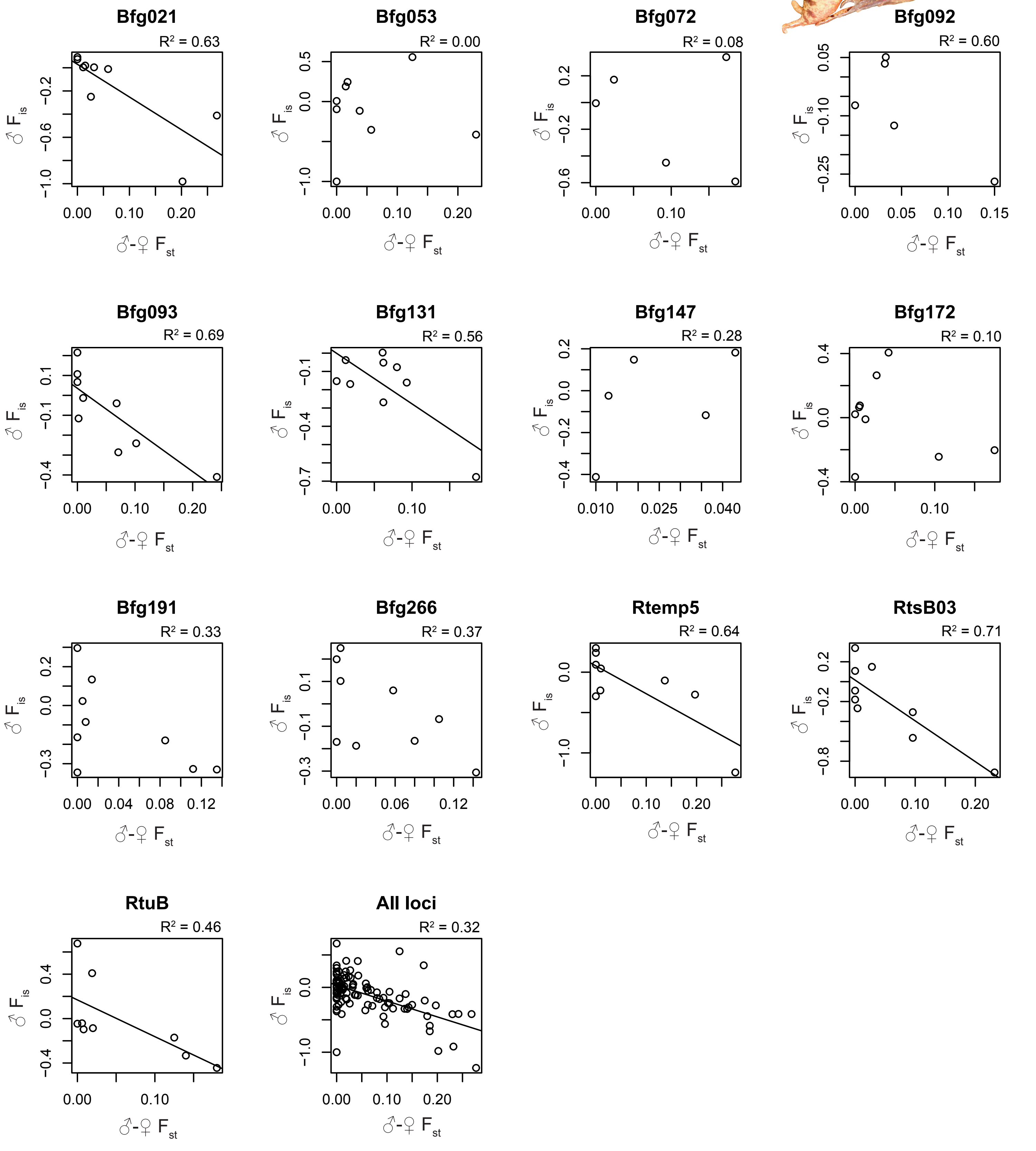

Supplement: Figure S2 [file peerj-05-3207-s003.pdf]
